# An Improved and Simplified Propagation System for Pollen-Free Sugi (*Cryptomeria japonica*) *via* Somatic Embryogenesis

**DOI:** 10.3389/fpls.2022.825340

**Published:** 2022-02-08

**Authors:** Tsuyoshi E. Maruyama, Momi Tsuruta, Saneyoshi Ueno, Kiyohisa Kawakami, Yukiko Bamba, Yoshinari Moriguchi

**Affiliations:** ^1^Department of Forest Molecular Genetics and Biotechnology, Forestry and Forest Products Research Institute, Tsukuba, Japan; ^2^Verde Co., Ltd., Toyohashi, Japan; ^3^Niigata Prefectural Forest Research Institute, Murakami, Japan; ^4^Graduate School of Science and Technology, Niigata University, Niigata, Japan

**Keywords:** Cupressaceae, embryogenic cells, pollen-free plants, pollinosis, somatic embryogenesis, somatic seedlings, clonal propagation, marker-assisted selection

## Abstract

Sugi (Japanese cedar, *Cryptomeria japonica*) is the most important forestry tree species in Japan, covering 44% of the total artificial forest area. Large amounts of pollen released from these forests each spring cause allergic reactions in approximately 40% of the population, which are a serious social and public health problem in Japan. As a countermeasure, there is an urgent need to reforest using male-sterile plants (MSPs; pollen-free plants); however, the production of MSPs *via* conventional methods is inefficient, time consuming, and requires considerable resources in terms of labor and space. In the present paper, we described an improved and simplified methodology for the efficient propagation of pollen-free Japanese cedar, combining the use of genetic markers (marker-assisted selection or marker-aided selection) for the early selection of male-sterile genotypes and the use of somatic embryogenesis (SE) for the clonal mass propagation of seedlings. We describe all the stages involved in the production process of somatic seedlings. Our results demonstrated that this methodology easily and efficiently produces MSPs with a discrimination rate of 100% in a short period of time. Production of 243.6 ± 163.6 cotyledonary embryos per plate, somatic embryo germination, and plantlet conversion frequencies of 87.1 ± 11.9% and 84.8 ± 12.6%, respectively, and a 77.6 ± 12.1% survival rate after *ex vitro* acclimatization was achieved. Moreover, we also describe an easy method for the collection of somatic embryos prior to germination, as well as an efficient and practical method for their storage at 5°C. Finally, a representative schedule for the propagation of pollen-free sugi somatic seedlings is presented as a reference for practical uses. This methodology will definitively help to accelerate the production of *C. japonica* MSPs across Japan.

## Introduction

Sugi (Japanese cedar, *Cryptomeria japonica* (Thunb. ex L.f.) D.Don, Cupressaceae) forests have been greatly exploited over the past 1,000 years; the artificial planting of sugi is estimated to have begun more than 500 years ago (Ohba, 1993), including clonal forestry *via* cuttings, which began in the beginning of the 15th century (Toda, 1974). Now, Japanese cedar is the most important forestry species in Japan, covering 44% of the total artificial forest and accounting for approximately 4.5 million ha (Forestry Agency, 2020). In contrast to its commercial importance, the large amounts of pollen released from sugi forests each spring cause allergic reactions, which are a serious social and public health problem in Japan; an estimated 40% of people living in Japan suffer from allergic rhinitis caused by sugi pollen (Matsubara et al., 2020), resulting in significant economic losses each year. In this context, the use of male-sterile plants (MSPs; pollen-free plants) in reforestation is one of the most effective countermeasures against pollinosis. However, the production of MSPs is associated with several problems that limit its practical implementation; one of these problems is the scarcity of material used for breeding. Since the probability of encountering male-sterile individuals in sugi forests is estimated to be one in several thousand (Igarashi et al., 2006), the discovery of new breeding material requires enormous effort. To date, only 23 male-sterile sugi individuals have been reported across the entire country (Saito, 2010); the majority of these male-sterile trees possess *MALE STERILITY 1* (*MS1*), a principal causative gene of male sterility in sugi (Moriguchi et al., 2020), which is associated with a developmental abnormality in the pollen tetrad period caused by one recessive allele *ms1* (Taira et al., 1999; Miura et al., 2011). At present, seeds for the propagation of *C. japonica* MSPs are produced by artificial crossing between a male-sterile tree (*ms1*/*ms1*), as a seed parent, and a pollen donor (an elite tree selected based on growth performance and morphological traits) with heterozygous sterile allele (*Ms1*/*ms1*; Maruyama et al., 2020; Tsuruta et al., 2021a).

Another challenge is the propagation and selection of pollen-free seedlings. Currently, MSPs are discriminated from the resulting seedlings after the application of gibberellin (GA_3_), which induces early flowering in sugi (Shidei et al., 1959; Nagao, 1983). After the induction of flowering, approximately half of the propagated seedlings that are not pollen-free due to Mendelian inheritance are unusable fertile individuals that must be discarded, despite having been in the nursery for 2 to 3 years leading up to the selection test. In this conventional method, the seedling production efficiency is extremely low and requires many resources in terms of time, labor, and space. In this sense, our goal is to establish efficient and easy protocols to produce excellent Japanese cedar MSPs in a short period of time. Our strategy is based on a combination of the use of genetic markers (marker-assisted selection or marker-aided selection, MAS) for the early discrimination of male-sterile genotypes of embryogenic cell (EC) lines (ECLs) and the use of somatic embryogenesis (SE) for the clonal mass propagation of seedlings. The development of this novel technique for the efficient production of sugi MSPs was first reported by our research group (Maruyama et al., 2020). Then, we released improved methodologies for SE initiation (Maruyama et al., 2021a), somatic embryo production, and somatic plant regeneration (Maruyama et al., 2021b; Tsuruta et al., 2021b), as well as for the early effective selection of male-sterile ECLs (Hasegawa et al., 2021; Tsuruta et al., 2021a).

In the present report, we aim to further improve and simplify the methodology for the propagation of pollen-free Japanese cedar by describing a protocol for all the stages involved in the production process, including the preparation of SE initiation materials, the early discrimination of male-sterile genotype lines at the undifferentiated cell stage (EC) to produce MSPs at a rate of 100%, the production of somatic embryos, the germination and conversion of somatic plantlets, and the production of plug plants in greenhouse and containerized seedlings in the nursery. Moreover, we describe an easy method for collecting somatic embryos prior to germination, as well as an efficient and practical method for storage at 5°C without considerable loss of germination capacity. Finally, we present a representative schedule for the propagation of pollen-free sugi *via* SE (case study for the Niigata Prefecture, central region of Japan), as a reference for practical uses. We believe that this methodology provides basic and practical information that will accelerate the production of MSPs across the country and serve as a powerful tool to improve genetic molecular breeding technology for *C. japonica*.

## Materials and Methods

### Plant Material Preparation

**Figure 1:**
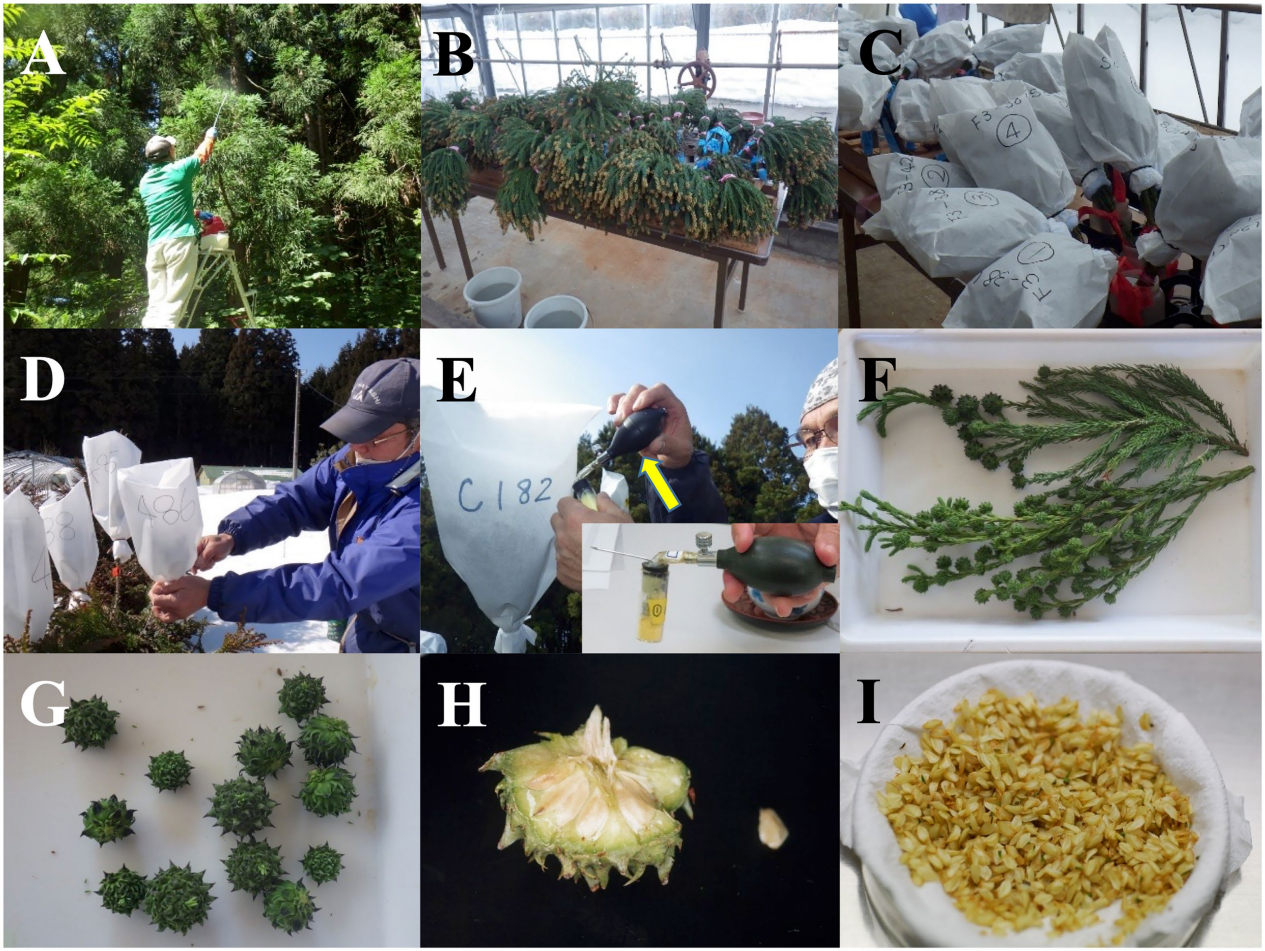
Plant material preparation for the propagation of pollen-free sugi (*C. japonica*) *via* somatic embryogenesis. **(A)** Application of gibberellin (GA_3_) to promote flowering. **(B)** Harvested branches with male flowers. **(C)** Branches with male flowers in bottles with water and covered with paper bags for pollen collection. **(D)** Covering of female flowers with pollination bags. **(E)** Injection of pollen into pollination bags with a pollen atomizer (yellow arrow; atomizer amplification is seen in the bottom right square). **(F)** Harvested branches with seed cones. **(G)** Collected seed cones. **(H)** Opened cone for seed extraction (cone size: 15 mm in diameter). **(I)** Seeds removed from cones (Petri dish size: 90 mm in diameter).

Plant materials for the propagation of pollen-free sugi were obtained by artificial crossings between a male-sterile tree (*ms1*/*ms1*) as a seed parent and a pollen donor with heterozygous sterile allele (*Ms1*/*ms1*; Maruyama et al., 2020; Tsuruta et al., 2021a). To ensure seed production, gibberellin (GA_3_) treatments were applied to the branches to promote flowering before the artificial crossings. The female flowers of mother trees in the seed orchard were artificially pollinated from March to April with previously collected donor pollen from elite trees that were selected based on their growth performance and morphological traits. Subsequently, the produced seed cones were collected in mid-July and their seeds containing immature zygotic embryos, mostly represented by early embryo stages equivalent to stages 5–6 on the scale described by Pullman and Buchanan (2003), were used as plant material for the initiation of SE. Details of the seed preparation process are described in Table 1 and Figure 1.

**Table 1:** Equipment, procedure, and season for each step of plant material preparation for the initiation of somatic embryogenesis in pollen-free sugi (*C. japonica*).

Step	Equipment	Procedure	Season
Application of gibberellin (GA_3_) to promote flowering	Gibberellin A_3_^1^ Sprayer Bucket Stepladder	Selected branches were sprayed or immersed into 100 mg L^−1^ GA_3_ solution for approximately 3–5 s. A booster of GA_3_ solution was applied approximately 1 week after the first treatment.	Late July and early August for male and female flowers, respectively
Harvest of branches with male flowers and pollen collection	Pruning shears Bottles Paper bags Stepladder Desiccator Silica gel	Harvested branches with male flowers were put in bottles with water and covered with paper bags. Branches were kept in a greenhouse (at about 25°C) until the male flowers opened and released pollen. Pollen collected in paper bags was kept in a desiccator with silica gel until use.	Late January to early February
Placement of pollination bags and artificial pollination	Pollination bags Selected pollen Pollen atomizer Stepladder Wire	Female flowers were covered with pollination bags (made with nonwoven material) before mid-February. Selected pollen was injected into the pollination bag using a pollen atomizer (about 0.2 g per bag) on sunny days from mid-March to early April (3 to 5 times).	Mid-February to early April
Collection of seed cones	Pruning shears Plastic trays Stepladder	Seed cones were separated from the harvested branches. Collected cones should be stored refrigerated (5°C) if not used immediately (up to about 4 to 8 weeks).	Mid-July
Extraction of seeds	Petri dishes Scalpels Paper tissue Ethanol	Seed cones were cleaned with tissue paper moistened with ethanol before they were opened with a scalpel. Seeds were removed from the cones and transferred to Petri dishes containing tissue paper moistened with water to avoid desiccation.	Mid-July

1 Gibberellin Kyowa Powder; Ministry of Agriculture, Forestry and Fisheries Registration No. 6007 (Kyowa Hakko Bio Co., Ltd., Tokyo, Japan).

### Culture Media and Conditions for SE

The culture media and conditions for the propagation of pollen-free sugi *via* SE are described in Supplementary Table S1 and Table 2, respectively. After autoclaving for 15 min at 121°C, culture media containing basal salts at standard or modified concentrations from the original Embryo Maturation (EM) medium (Maruyama et al., 2000) were used, according to each step. In all steps, the plates were sealed with Parafilm®.

**Table 2:** Medium, culture condition, and culture duration for each stage in the propagation of pollen-free sugi (*C. japonica*) plants *via* somatic embryogenesis.

Culture stage	Medium^1^	Culture conditions	Duration
Embryogenic culture initiation	EM-1^1^	Dark at 25°C; 90 × 15 mm Quad-plates (30–35 ml medium/plate); 3 megagametophytes/well (12/plate).	3–12 weeks
Embryogenic cell maintenance/proliferation	EM-2^1^	Dark at 25°C; 90 × 15 mm Quad-plates (30–35 ml medium/plate); 3 embryogenic cell masses/well (12/plate).	2-week intervals
Maturation of somatic embryos	EM-3^1^	Dark at 25°C; 90 × 20 mm Mono-plates (30–40 ml medium/plate); 3–5 EC masses/plate.	6–10 weeks
Storage of somatic embryos	EM-3^1^	Dark at 5°C; Plates with cotyledonary embryos from the maturation stage were stored inside Ziploc^®^ freezer bags.	up to 24 months
Somatic embryo germination and plantlet conversion	EM-4^1^	Light at 25°C; 16-h photoperiod (about 2,500–3,500 lx); 90 × 20 mm Mono-plates (30–40 ml medium/plate); about 50–150 coyledonary embryos/plate.	8–12 weeks
*Ex vitro* acclimatization of somatic plantlets	Plant plug^2^	Light: up to about 10,000 lx; Temperature: 15–35°C; Relative humidity: 30–70%; Mist watering: 20–30 s/every 20–30 min during the daytime; Manual watering: once per day, normally in the morning.	3–4 weeks
Growth of somatic plug plants after acclimatization	Plant plug^2^	Light: up to about 25,000 lx; Temperature: 15–35°C; Relative humidity: 30–70%; Manual watering: once per day, normally in the morning; Fertilization: 0.15% (w/v) plant-food solution^3^ once per week.	9–10 weeks
Containerized somatic seedlings	Seedling container^4^	Natural nursery conditions; Potting soil: mixture of Cocopeat-old (80%) and Kanuma soil (20%) with a supplement of N-P-K (in mg L^−1^: 500, 900, and 750, respectively)^5^; Sprinkler watering: 15 min once per day (15 min twice per day in the summer); Additional fertilization: 0.4% (v/v) plant-food solution^6^ once per week from July to September; Seedling disinfection: application of a solution of Bordeaux mixture^7^ once every 2 weeks from June to September.	9–16 months^8^

1 See Supplementary Table S1.

2 200 holes per tray Jiffy Preforma^®^ plant plugs (Sakata Seed Co., Yokohama, Japan).

3 Peter’s 20-20-20 soluble fertilizer (The Hyponex Co., Inc., Hyponex Japan, Osaka, Japan).

4 JFA-150 (150 ml; 8 × 5 cavities per tray) seedling containers (National Federation of Forest Seedling Cooperatives, Tokyo, Japan).

5 Potting soil for container seedlings (Top Co., Ltd., Osaka, Japan).

6 Hyponex 6-10-5 liquid fertilizer (The Hyponex Co., Inc., Hyponex Japan, Osaka, Japan).

7 Bordeaux mixture IC-66D; Ministry of Agriculture, Forestry and Fisheries Registration No. 18645 (Inoue Calcium Co., Ltd., Kochi, Japan).

8 Niigata Prefecture case study.

### Embryogenic Culture Initiation

The whole megagametophyte, containing the immature zygotic embryo, was used as the initial explant for SE initiation. The seeds were surface sterilized with 1% (w/v) sodium hypochlorite solution (Wako Pure Chemical, Osaka, Japan) for 15 min, then rinsed three times for 5 min each with sterile distilled water. Then, the seed coat was removed with sterile scalpels and forceps to aseptically isolate the megagametophytes from the seeds under a dissecting microscope. Explants were placed horizontally into initiation EM-1 medium (Supplementary Table S1) and cultured under the conditions described in Table 2. The induction of ECs from the explants was monitored up to 12 weeks of culture. The ECLs were noted if initiated ECs proliferated after the first subculture.

### Maintenance and Proliferation of ECLs

Proliferated ECs were collected with forceps from the initiation EM-1 medium and transferred to a maintenance/proliferation medium (EM-2, Supplementary Table S1). Subsequently, the tissues of established ECLs were regularly subcultured at 2-week intervals. EC masses of about 25–50 mg of fresh weight (FW) were cultured under the conditions described in Table 2.

### Discrimination of Male-Sterile ECLs by MAS

Using 5 to 10 mg of the maintained ECs, DNA was extracted using InstaGene Matrix (Bio-Rad, Hercules, CA, United States). The *MS1* genotype of the ECL was determined using PCR amplification of the marker directly developed for the gene that causes male sterility (Tsuruta et al., 2021a), followed by electrophoresis on 1.5% agarose gel. Each reaction condition is described in Table 3.

**Table 3:** Procedures and reaction conditions for a simplified method for the discrimination of pollen-free sugi (*C. japonica*) embryogenic cell lines.

Step	Equipment	Procedure	Reaction time
DNA extraction	Centrifuge Dry bath heater Vortex mixer Magnet stirrer	5 to 10 mg of ECs added to 200 μl of extraction solution with Chelex resin^1^ and vortexed for 10 s. Following 8 min of incubation at 100°C and vortexing for 10 s, centrifugation was performed at 13,000 rpm for 1 min.	Within 15 min per sample
PCR amplification of the DNA marker designed on the *MS1* gene itself	Thermal cycler Ice bath	Ten microliters of PCR reaction mixture contained 1 μl of DNA extract, 3 μl of 2× QIAGEN Multiplex PCR Master Mix (Qiagen), and 0.2 μM each primer.^2^ PCR cycling comprised of denaturation at 94°C for 15 min, followed by 35 cycles of 94°C for 15 s, 60°C for 30 s, and 72°C for 30 s, with a final extension for 5 min at 72°C.	Approx. 2 h
Electrophoresis and taking gel images	Gel electrophoresis system (such as Mupid) Gel imaging system	PCR products were separated by 1–1.5% agarose gel electrophoresis (30–40 min), then photographs of the stained gels were taken.	Approx. 1 h

1 InstaGene matrix (Bio-Rad, Hercules, CA, United States).

2 Primer sequences described in Tsuruta et al. (2021a).

### Maturation of Somatic Embryos

**Figure 2:**
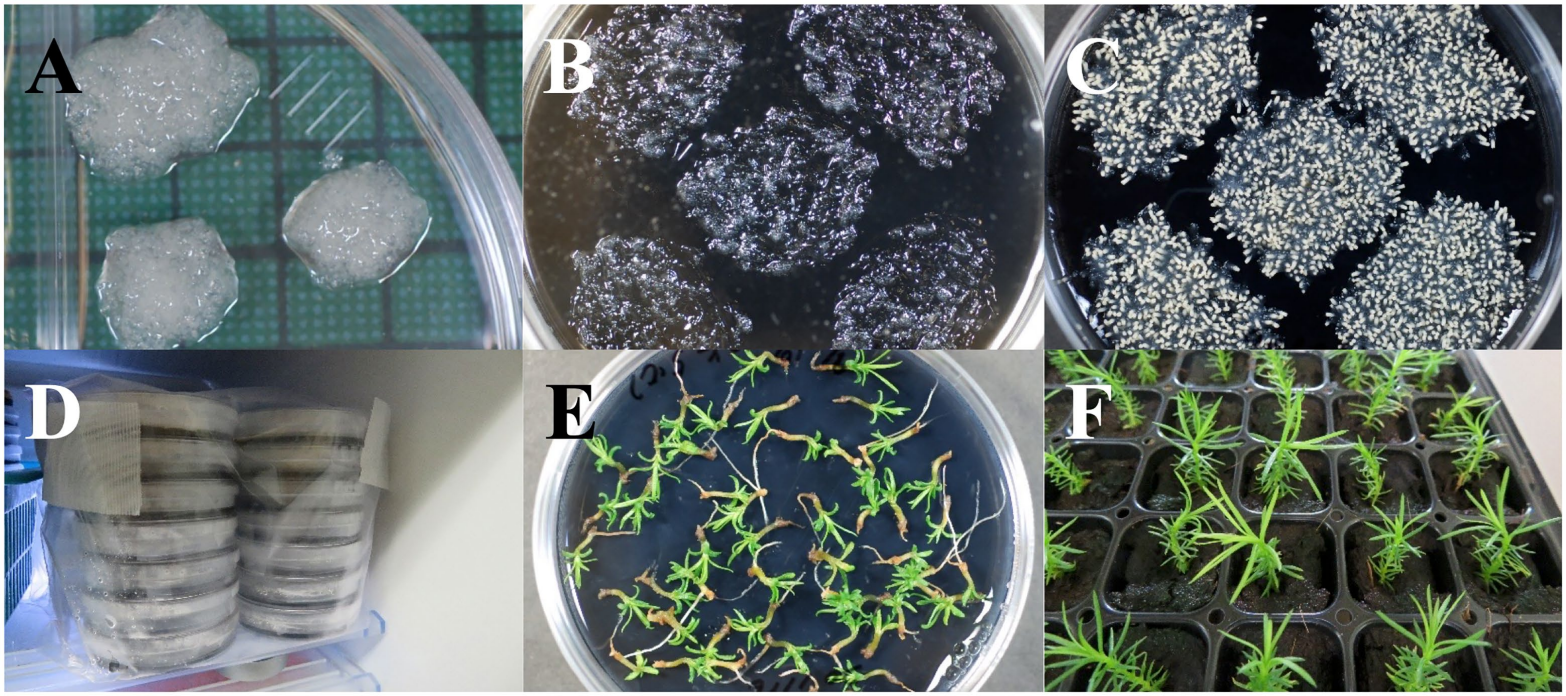
Maturation and storage of somatic embryos of pollen-free sugi (*C. japonica*). **(A)** Embryogenic cell proliferation after 2 weeks of culture. **(B)** Proliferated embryogenic cells homogeneously dispersed into the maturation medium. **(C)** Somatic embryo maturation after approximately 6 weeks of culture. **(D)** Storage of somatic embryos at 5°C. **(E)** Germination of somatic embryos after 2 years of storage at 5°C. **(F)** Regenerated somatic plantlets from 2 year stored embryos transferred into plant plugs for *ex vitro* acclimatization. Diameter of plates: 90 mm; size of plant plugs: 23 × 23 × 38 mm.

For somatic embryo maturation, three to five proliferated EC masses (about 100 mg FW each) from the maintenance/proliferation medium (Figure 2A) were transferred and homogeneously dispersed by forceps into a plate containing maturation EM-3 medium (Supplementary Table S1). Each mass was dispersed on a surface equivalent to a circle with a diameter of approximately 2.5–3.0 cm (Figure 2B). Plates were sealed and cultured under the conditions described in Table 2.

### Storage of Somatic Embryos

After the end of the maturation stage, culture plates containing both maturation medium and mature embryos (Figure 2C) were sealed with two more layers of Parafilm® then stored in Ziploc® freezer bags at 5°C (Figure 2D). The viability of stored somatic embryos was monitored at different storage periods (0, 6, 12, and 24 months) *via* germination tests on EM-4 medium (Figure 2E; Supplementary Table S1). Regenerated somatic plantlets were acclimatized in plant plugs (Figure 2F), as described in the following *ex vitro* Acclimatization section.

### Somatic Embryo Germination and Plantlet Conversion

**Figure 3:**
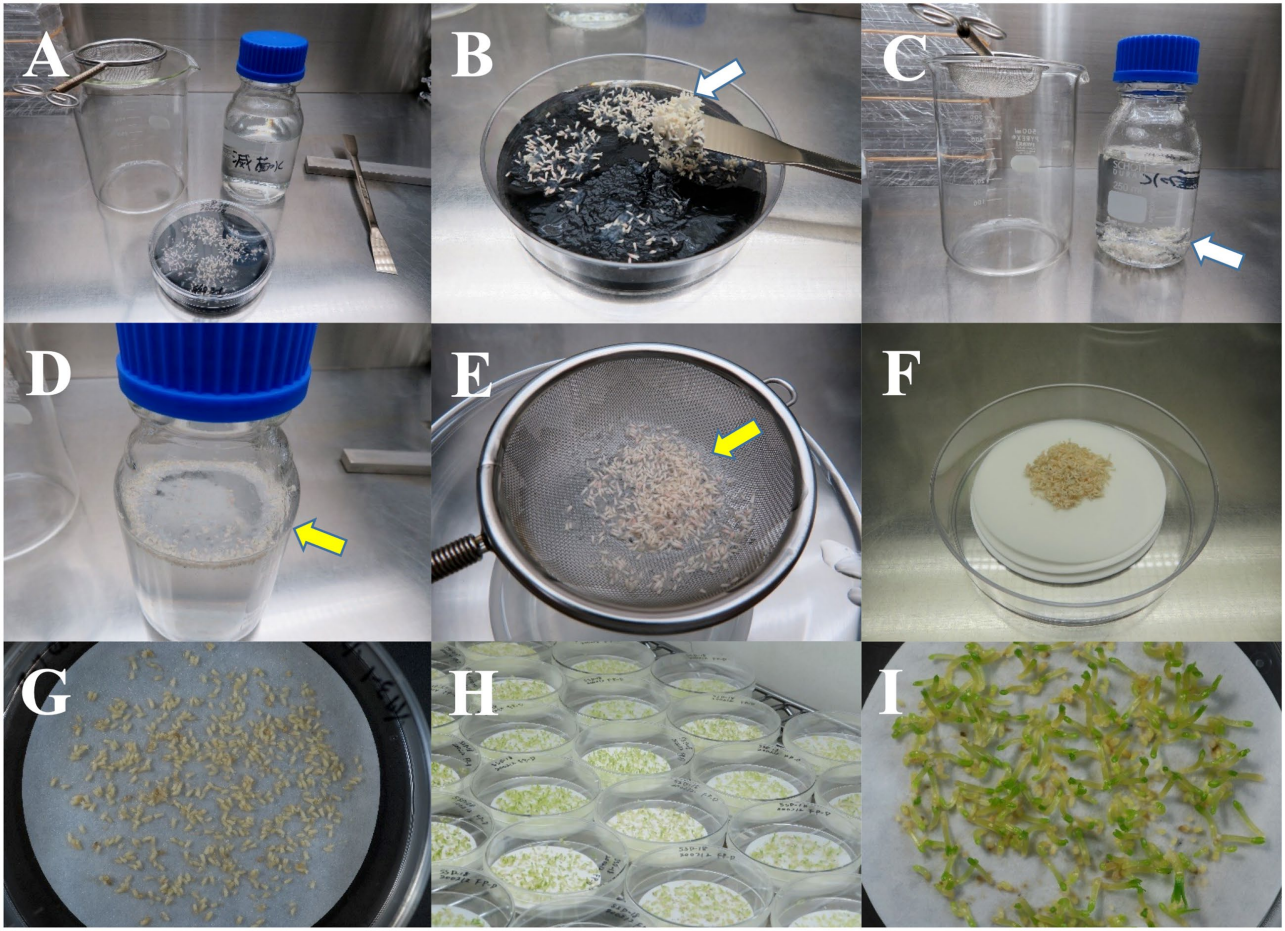
Collection of pollen-free sugi (*C. japonica*) somatic embryos from the maturation medium. **(A)** Materials used for the collection of matured somatic embryos. **(B)** Clusters of somatic embryos and embryogenic cells (ECs; white arrow) collected using a spatula, then transferred into a flask with sterile distilled water **(C)**. **(D)** Embryos (yellow arrow) separated from the adhering proliferated ECs after vigorous shaking of the flask and then collected on a tea strainer **(E)**. **(F)** Collected embryos transferred into filter papers inside an empty plate. **(G)** Embryos placed into filter paper, then incubated inside empty plates for about 2 weeks before transfer to the germination/conversion medium **(H,I)**. Diameter of filter paper: 70 mm.

Somatic embryos were collected from the maturation medium with a spatula and transferred into a flask containing sterile distilled water. After several vigorous shaking episodes to separate the adhering proliferated ECs, the somatic embryos were collected on a tea strainer and then transferred into filter papers inside empty plates (Figures 3A–F). After dispersing the embryos into filter paper with forceps (Figure 3G), the plates were sealed and incubated at 25°C for approximately 2 weeks (Figures 3H,I) for remove the water remaining in filter papers before being transferred to the germination/conversion EM-4 medium (Supplementary Table S1) and cultured under the conditions described in Table 2. The emergence of roots (germination) and the emergence of both roots and epicotyl (plantlet conversion) were recorded after 8 weeks of culture.

### *Ex vitro* Acclimatization and Growth of Acclimatized Somatic Plants

**Figure 4:**
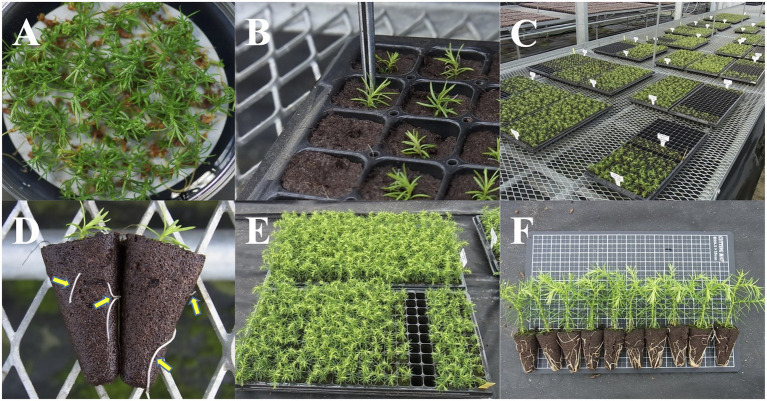
*Ex vitro* acclimatization and growth of acclimatized pollen-free sugi (*C. japonica*) somatic plants. **(A)** *In vitro* regenerated somatic plantlets. **(B)** Transfer of somatic plantlets into plant plugs. **(C)** Plug plants kept inside the greenhouse under mist watering control. **(D)** Acclimatized somatic plants showing roots (yellow arrows) emerging from the plugs. **(E,F)** Growth and appearance of somatic plants approximately 3 months after their transfer into plant plugs. Size of plant plugs: 23 × 23 × 38 mm.

After 12 weeks of culture on the germination/conversion medium, regenerated somatic plantlets (Figure 4A) were gently transferred into plant plugs with forceps (Figure 4B), then kept in the greenhouse under mist watering control for *ex vitro* acclimatization (Figure 4C). During the acclimatization period, both mist and manual watering were applied. After the complete acclimatization of somatic plants (after approximately 3–4 weeks when roots had emerged; Figure 4D), mist watering was halted; irrigation continued with manual watering, usually once per day in the morning, and with the application of a plant-food solution once per week. Acclimatized plants were kept in the greenhouse for an additional 9–10 weeks before being transplanted into seedling containers (Figures 4E,F). The daylight intensity over the plug plants was regulated by a shade-net covering. Additional details of the *ex vitro* acclimatization conditions are described in Table 2.

### Containerized Somatic Seedlings

**Figure 5:**
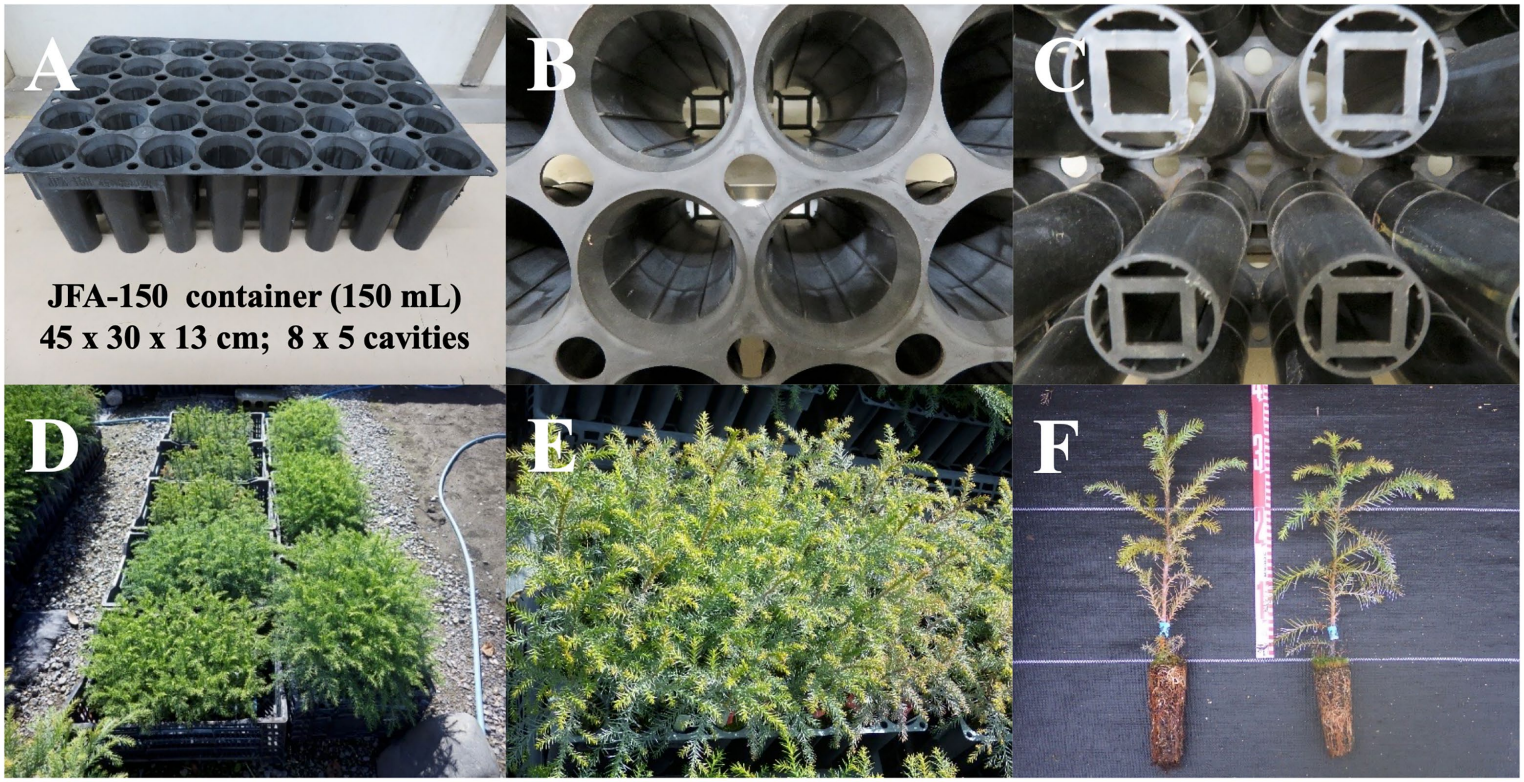
Containerized somatic seedlings of pollen-free sugi (*C. japonica*). **(A)** JFA-150 container. **(B)** View of the ribs on the inner wall of the cavity. **(C)** Bottom of the container. **(D)** An example of the growth status of containerized somatic seedlings derived from different embryogenic cell lines. **(E,F)** Containerized seedlings with heights of more than 30 cm, suitable for planting in the field.

Plug plants with heights greater than 5 cm (Figure 4F) were transplanted into seedling containers (Figures 5A–C) filled with a potting soil mixture fertilized with N-P-K and kept in the nursery under natural outdoor conditions (Figures 5D,E). Seedlings were irrigated daily for 15 min *via* sprinkler watering in the morning, and for 30 min in the summer season (15 min in the morning and 15 min in the evening). From July to September, once per week, additional fertilization with a plant-food solution was applied. To prevent pests and diseases, seedlings were fumigated with a Bordeaux mixture solution once every 2 weeks from June to September. Details of the containerized somatic seedling conditions are described in Table 2.

### Statistical Analysis

Cell line differences in terms of SE efficiencies (i.e., EC proliferation, somatic embryo maturation, germination, and plantlet conversion) and the growth of somatic seedlings (acclimatized somatic plants and containerized somatic seedlings) were tested using generalized linear models (GLMs) with cell line as the explanatory variable. For germination capacity after storage, we constructed a GLM with storage period as the explanatory variable; for these, Gaussian, Poisson, and binomial distributions were used for the error distributions of the proliferation and somatic seedling growth, maturation efficiency, and germination and conversion rates, respectively. For the analyses with cell line as the explanatory variable, the post-hoc analyses for pairwise comparisons of each cell line were conducted using the R package “multicomp” (Bretz et al., 2010) with BH adjustment.

## Results

### Embryogenic Culture Initiation

Initiation frequencies from seed collection in mid-July varied from 0.9 to 49.9%, depending on the seed family and collection year; the average initiation frequency was 26.0% (SD ± 18.9) for the seven collections, including six different evaluated seed families (Table 4). Observations of the response of the initial explants to induce ECs, monitored for up to 12 weeks of culture, revealed that the extrusion of ECs from the explants started after approximately 2 weeks of culture, with evident proliferation at 3–4 weeks after extrusion. Although some explants responded after 9 weeks of culture, the majority did so between the 2nd and 8th week.

**Table 4:** Embryogenic cell induction from different pollen-free sugi (*C. japonica*) seed families.

Seed family	Seed family code	Collection year	Number of explants tested	Explants with embryogenic cell induction	Induction rate (%)
♀ “Toyama-funen 1” × “Ohara 2” ♂ “Suzu 2”	TOS	2013	1,228	244	19.9
♀ “Shindai 3” ♂ “Suzu 2”	SSD	2016	420	59	14.0
♀ “Shindai 3” ♂ “Suzu 2”	S	2017	383	191	49.9
♀ “Toyama-funen 1” ♂ “Toyama-funen 1” × “Nakakubiki 4”	TON	2016	971	9	0.9
♀ “Fukushima-funen 1” ♂ “Shindai 3” × “Kamikiri 2”	FSKam	2017	516	62	12.0
♀ “Fukushima-funen 1” ♂ ‘Ōi 7’	FO7	2017	636	259	40.7
♀ “Fukushima-funen 1” ♂ “Shindai 3” × “Kashiwazaki-shi 3”	FSKas	2017	429	191	44.5
				Mean (SD)	26.0 (18.9)

### Maintenance and Proliferation of ECLs

**Figure 6:**
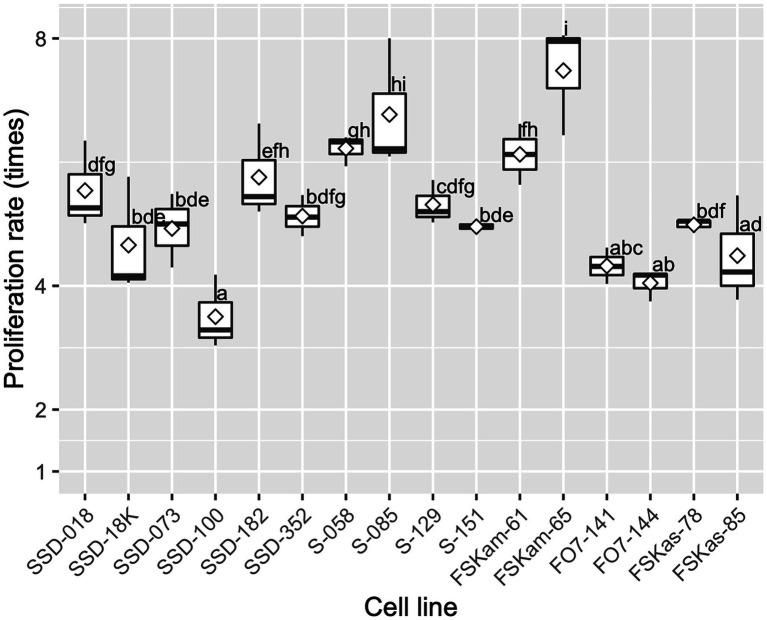
Embryogenic cell proliferation efficiency for different cell lines of pollen-free sugi (*C. japonica*) derived from four seed families (proliferation rate average ± SD for each cell line). SSD: “Shindai 3” × “Suzu 2” (2016); S: “Shindai 3” × “Suzu 2” (2017); FSKam: “Fukushima-funen 1” × (“Shindai 3” × “Kamikiri 2”); FO7: “Fukushima-funen 1” × “Ōi 7”; and FSKas: “Fukushima-funen 1” × (“Shindai 3” × “Kashiwazaki-shi 3”). The open diamond in the boxplot represents the mean. The different letters indicate significant differences among the cell lines (*p* < 0.05, pairwise comparison with BH adjustment).

After 2 weeks of culture on the maintenance/proliferation EM-2 medium, the 16 ECLs had an average proliferation rate of 5.3 ± 1.0 times the initial FW; this rate varied, depending on the ECL, from 3.5 to 7.5 times the initial FW of ECs (Figure 6). These differences in proliferation capacity between ECLs remained generally the same in subsequent routine subcultures.

### Discrimination of Male-Sterile ECLs by MAS

Our simplified DNA extraction and DNA marker diagnosis methods allowed us to genotype *MS1* with only 15 min of extraction time per sample and a single PCR and electrophoresis (Supplementary Figure S1). Since the *MS1* gene itself was used as a DNA marker, based on the genotyping results, pollen-free ECLs could be selected with 100% accuracy.

### Maturation and Storage of Somatic Embryos

**Figure 7:**
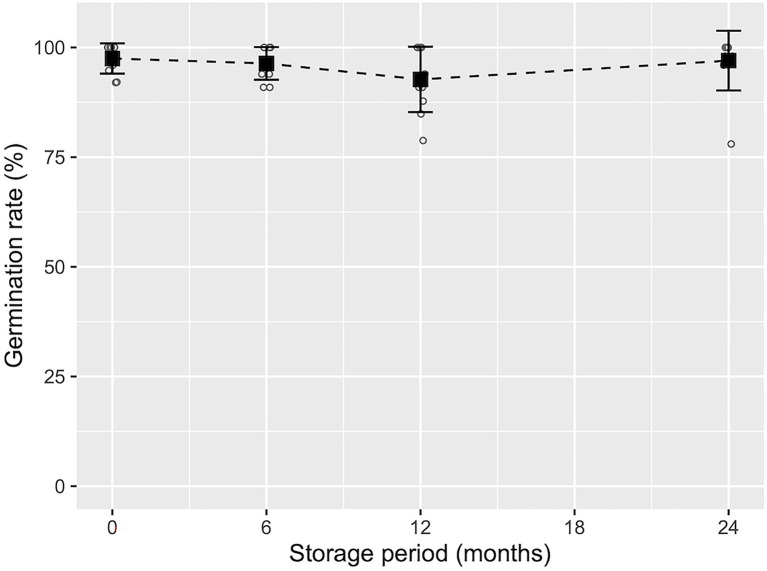
Germination frequency of pollen-free sugi (*C. japonica*) somatic embryos derived from the SSD-018 embryogenic cell line after different storage periods. The average germination frequency (black square) with error bars (±SD) at each storage period is shown above the measurements (white circles).

Development of cotyledonary embryos was observed most frequently 6–8 weeks after the transfer of ECs to maturation medium (Figures 2C, 3B). As shown in Supplementary Figure S2, although the maturation efficiency differed among the ECLs, the induction of cotyledonary embryos was achieved in all lines tested. The average number of induced mature somatic embryos was 243.6 ± 163.7, with a range of 25.0 to 513.8 embryos per plate. We also investigated the effects of short- to medium-term cold storage (5°C) for the maintenance of the germination capacity of somatic embryos. Across the different tested storage periods, the average germination rate was 92.7 ± 7.5%, with a range of 78.8 to 100%. No significant decrease in the germination capacity of somatic embryos was observed after the different storage periods (*p* > 0.05, GLM, Figure 7).

### Somatic Embryo Germination and Plantlet Conversion

The germination of mature somatic embryos began approximately 1–2 weeks after transfer to the germination/conversion EM-4 medium; in most cases, plantlet conversion was achieved after approximately 6 weeks of culture. As shown in Supplementary Table S2, embryo germination and plantlet conversion varied across the ECLs from 52.1 to 97.5% and 47.7 to 96.6%, respectively. The average germination and conversion rates for the 19 evaluated ECLs were 87.1 ± 11.9% and 84.8 ± 12.6%, respectively.

### *Ex vitro* Acclimatization and Growth of Acclimatized Somatic Plants

**Figure 8:**
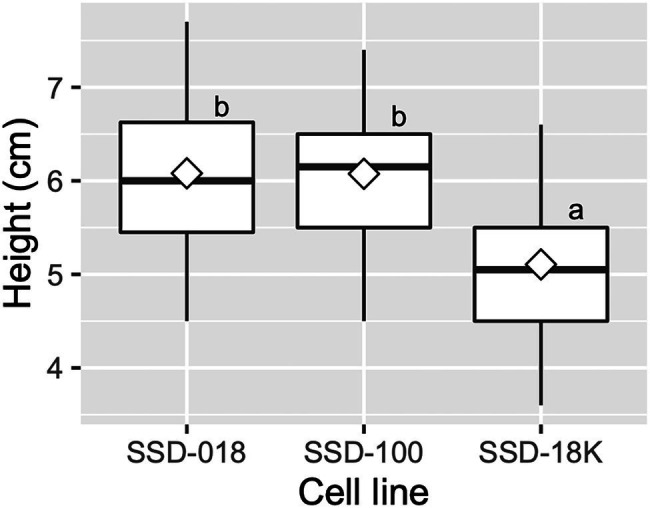
Growth of acclimatized somatic plants of pollen-free sugi (*C. japonica*) 3 months after their transfer into plant plugs. The open diamond in the boxplot represents the mean. The different letters indicate significant differences among the cell lines (*p* < 0.05, pairwise comparison with BH adjustment).

Across somatic plant lines after acclimatization in plant plugs, the average survival rate was 77.6 ± 12.1%, ranging from 63.9 to 87.0%. Across the somatic plant lines 3 months after their transfer into plant plugs, the average growth in height was 5.8 ± 0.6 cm and the average range was 5.1–6.1 cm (Figures 4F, 8).

### Containerized Somatic Seedlings

**Figure 9:**
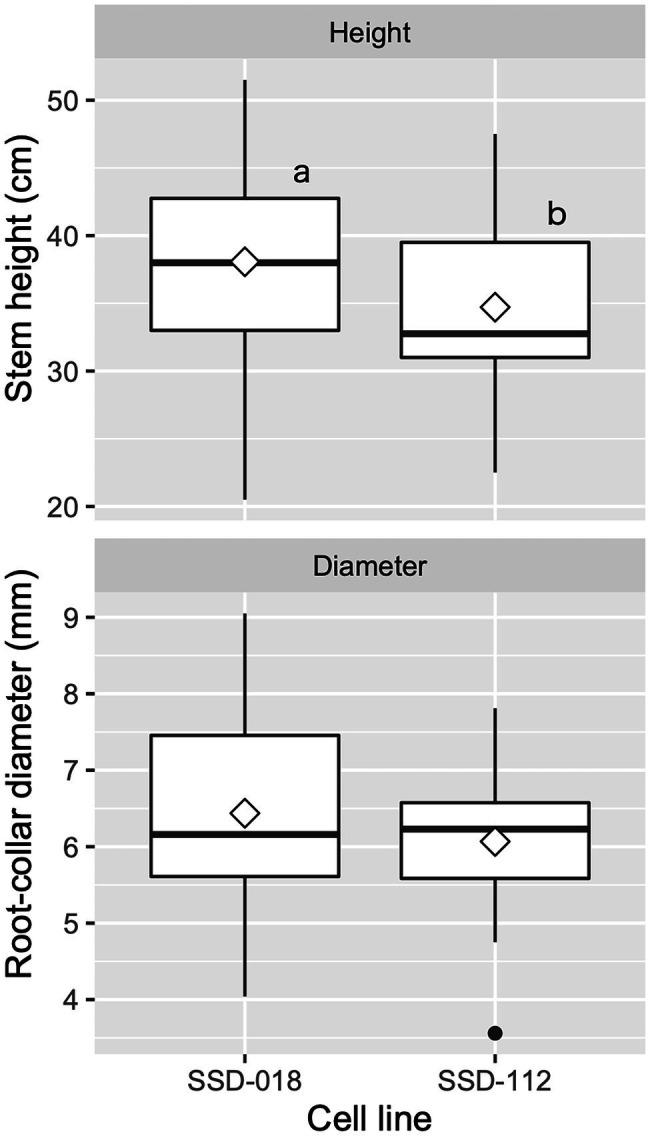
Growth of containerized somatic seedlings of pollen-free sugi (*C. japonica*) in Niigata Prefecture (central region of Japan) before being planted in the field. The open diamond and the dot represent the mean and outlier, respectively. The different letters indicate significant differences among the cell lines (*p* < 0.05, pairwise comparison with BH adjustment).

After the transfer of the plug plants into seedling containers during the spring season, they were cultivated in the nursery until they reached a height of 30 cm or more (Figures 5E,F). The average survival rate was 91.7 ± 7.4%, ranging between 89.6 and 100.0% across somatic seedling lines. Figure 9 shows the growth of containerized somatic seedlings in Niigata Prefecture before field planting. The average height/basal diameter for somatic seedling lines SSD-018 and SSD-112 was 38.1 cm/6.4 mm and 34.7 cm/6.1 mm, respectively.

## Discussion

### Somatic Embryogenesis Initiation

Although SE from the vegetative material of selected adult trees is the ideal method for clonal propagation in all practical situations, like in other conifers, the initiation of SE in sugi remains limited to the use of seeds. Despite our considerable efforts to reproduce the successful SE initiation protocols for the adult trees of white spruce and some pines (Park et al., 2010; Klimaszewska et al., 2011; Malabadi et al., 2011; Teixeira da Silva and Malabadi, 2012), we have yet to achieve positive results in terms of inducing ECs from adult vegetative material in sugi and other Japanese conifers (Maruyama T.E., unpublished). Therefore, seeds remain the only starting material that can be used to initiate SE in sugi. Seed production in sugi is effectively promoted by stimulating flowering with the application of gibberellin (GA_3_) during the summer season, mainly in late July and with a booster application in early August (Table 1; Figure 1). Similarly, although using different concentrations, forms of application, and types of gibberellin, this treatment is also reported to stimulate flowering in other conifers, including pines, cypresses, and Douglas firs (Hashizume, 1985; Kon and Koyama, 2000; Kato et al., 2015; Kong et al., 2021).

As detailed in our previous report (Maruyama et al., 2021a), sugi SE initiation from male-sterile seed families is influenced by several factors, mainly related to the initial explants, timing of collection, genotypes, and culture conditions for the induction of ECs. Although SE initiation is practically feasible from mid-June to late July (Maruyama et al., 2000, 2014), the highest initiation frequencies were achieved using whole megagametophytes as initial explant isolates from seeds collected in mid-July; this result is consistent with the reported initiation frequencies from open-pollinated (Ogita et al., 1999; Taniguchi and Kondo, 2000) and artificially pollinated (Taniguchi et al., 2020) male-fertile seed families. The results achieved throughout our experiments proved that, even though the initial explant, collection time, and culture conditions played an important role, the genotype of the plant material was the most influential factor for the initiation of SE. As shown in Table 4, the initiation rates varied considerably depending on the genotype of the initial explant. Similarly, in accordance with our results, the initiation of SE in several conifers is genetically controlled, perhaps independently of other traits (Klimaszewska et al., 2007). Since the most important role of conifer SE is the deployment of genetically tested tree varieties integrated in tree improvement programs (Park et al., 2006), improvement in SE initiation, with respect to capturing as many genotypes as possible to enhance the representation of families, is important for developing varietal lines and is also fundamental for the management of genetic diversity (Hargreaves et al., 2009). Therefore, the screening of responsive seed family genotypes *via* the multiyear selection of appropriate parents is necessary to increase the initiation rate of SE.

On the other hand, as reported for a number of species, extruded ECs did not always proliferate or result in the establishment of an ECL (Maruyama and Hosoi, 2019). Thus, initial extrusion from an explant should be distinguished from stable continuous growth, when assessing success rates (Klimaszewska et al., 2007). In our experiments, the induction of stable ECLs was used as the criterion for evaluating SE initiation capacity. Subsequently, the continuous proliferation of stable ECLs was promoted *via* culturing on medium supplemented with 2,4-dichlorophenoxyacetic acid and 6-benzylaminopurine; notwithstanding that the proliferation rate significantly varied across ECLs (Figure 6), the maintenance/proliferation EM-2 medium (Supplementary Table S1) described in this methodology was able to support the growth of ECs for several years using 2-week subculture routines. This medium has also been successfully used for the maintenance and proliferation of ECLs in other Japanese conifers (Maruyama et al., 2002, 2005a,b,c, 2007, 2011, 2012; Hosoi and Maruyama, 2012).

### Discrimination of Male-Sterile ECLs by MAS

By combining simple DNA extraction with the InstaGene matrix and diagnosis with the marker developed in the *MS1* gene itself, the MAS of a pollen-free sugi line was achieved in the ECs of the early SE stage. This saved a significant amount of labor and space that is otherwise wasted on male-fertile samples, which must be maintained until a decision could be made based on gibberellin-induced flowering. Using the simple extraction method described here, DNA could be extracted from as little as 5 mg of ECs. The application of the InstaGene matrix to plants has been previously reported by HwangBo et al. (2010). We have also shown that this simple extraction method is very effective for coniferous ECs. Although the use of this extraction method can be extended to other materials like adult leaves, extraction is more difficult in later culture materials, such as cotyledonary embryos, than in ECs (Tsuruta et al., 2021a).

DNA marker discrimination can also be made more stable. The previously used allele-specific PCR markers for genotype identification require a reaction for each allele, and sometimes show ambiguous bands (Hasegawa et al., 2020; Maruyama et al., 2020). In addition, recombination can be misidentified because previous MAS used markers linked to *MS1* (Ueno et al., 2019). Recent advances in genome sequencing technologies have made it possible to identify genes or mutations of objective traits, and gene-assisted selection (GAS) is also becoming possible for forest trees (Butcher and Southerton, 2007; Wilcox et al., 2007; Moriguchi et al., 2020). The determination of the *MS1* gene itself (Hasegawa et al., 2021) was a significant factor in achieving GAS in the MSP breeding of Japanese cedar.

### Maturation and Storage of Somatic Embryos

The efficient maturation of many genotypes, as well as the production of somatic plants with a high field performance, is the most important criteria for using SE protocols; this is important for mass production and breeding programs in addition to being a powerful tool for basic studies of molecular, genetic and physiological processes, and for genetic engineering. The genotype of the ECLs, media formulations, and culture conditions are some of the principal factors that influence SE (Tautorus et al., 1991; Stasolla et al., 2002; Teixeira da Silva and Malabadi, 2012; Egertsdotter, 2019; Gulzar et al., 2020; Izuno et al., 2020; Maruyama et al., 2021a). The results of our experiment indicate that, although mature somatic embryos were achieved in all ECLs, there were significant differences in maturation efficiencies across the evaluated genotypes (Supplementary Figure S2). This result confirms that somatic embryo maturation in sugi is controlled by the genetic origin of the ECL and highlights the importance of selecting a highly responsive champion cell genotype (we named it the “Yokozuna” cell genotype, referring to a grand champion sumo wrestler) to optimize the efficiency of SE protocols. Our improved protocol demonstrates that, by selecting genotypes, more than 500 high-quality somatic embryos (more than 1,000 embryos per gram FW) can be produced per plate. The ultimate goal of successful maturation is based on the fact that large-scale production of high-quality somatic embryos requires a high frequency of germination and can become a vigorous planting stock (Klimaszewska et al., 2007).

The short- to medium-term cold storage (5°C) of somatic embryos (presented in Figure 7) demonstrated that sugi embryos can be stored for at least 2 years without a significant decrease in germination capacity. Although cryopreservation is the ideal method for preserving genetic materials, it requires expensive equipment and special procedures. Moreover, since the objective of the short- to medium-term storage of somatic embryos is to provide flexibility in the seedling production schedule, allowing easy regulation of the optimal period for the transfer of somatic seedlings to the field, maintaining an embryo germination capacity of approximately 90% for 6 to 24 months is adequate to allow it to be implemented into our methodology. In accordance with our results, Torres-Viñals et al. (2004) reported more than 90% germination from the fresh isolated somatic embryos of grapevines after 30 days of storage at 4°C. Similarly, for the same species but using desiccated somatic embryos, Jayasankar et al. (2005) reported 90% plant conversion after 42 months of storage at 4°C. In contrast, Bornman et al. (2003) reported that cold storage at 4°C had a deleterious effect on the naked or encapsulated somatic embryo germination of *Picea abies* (L.) H.Karst. when stored for 1 month or longer. The somatic embryo storage protocol described in our methodology is easy, simple and only requires a conventional refrigerator for storage at 5°C, which will facilitate its application to practical uses.

### Somatic Embryo Germination and Plantlet Conversion

As shown in Supplementary Table S2, the high somatic embryo germination and conversion frequencies achieved in almost all of the evaluated ECLs demonstrated the high quality of the produced embryos. Even though the somatic embryo production of sugi was promoted by increasing the osmotic pressure of the medium *via* the addition of a large amount of polyethylene glycol (175 g L^−1^), the produced embryos germinated and converted into plantlets after transfer into a plant growth regulator-free medium, without the partial desiccation and/or cold postmaturation treatments that have been reported to be necessary in other conifers (Roberts et al., 1990; Lelu et al., 1995; Klimaszewska et al., 2007; Lara-Chavez et al., 2011; Liao and Juan, 2015; Pullman et al., 2016). In the present methodology, we also include a simple method for the collection of somatic embryos after maturation (Figure 3) and for their subsequent germination and plantlet conversion into the same medium (Figure 4A); we skip the growth step in culture flasks by transferring the regenerated plantlets directly from the germination plates to the plant plugs. This simplified procedure reduces labor and time compared to our previous protocols in which mature somatic embryos were collected individually with forceps to be transferred to the germination medium and then to another growth medium.

### *Ex vitro* Acclimatization and Containerized Somatic Seedlings

Male-sterile somatic plantlets were successfully acclimatized in plant plugs according to the procedures described in Table 2 and Figure 4. The plug plants grew well without signs of abnormal morphological appearance; although there were some differences in growth across the lines, most reached the appropriate height for transplantation into seedling containers after 9–10 weeks of cultivation. This result suggests that plant plugs of 200 holes per tray are appropriate to support the growth of sugi somatic plants. Although other larger-sized plant plugs (e.g., 128 and 72 holes per tray) also tested positively (data not shown), with a goal of reducing seedling production costs, we deemed it more appropriate to use smaller plant plugs (study in progress). After the transplantation of plug plants into containers, somatic seedlings were cultivated in the nursery. Currently, all over Japan, 150 ml (8 × 5 cavities per tray) and 300 ml (6 × 4 cavities per tray) type containers are the most popular containers used for seedlings and cuttings, respectively (FFPRI, 2021). However, this is the first report for Japanese cedar somatic seedlings. Therefore, due to the scarce information regarding tissue culture-derived plants and pollen-free sugi seedlings produced *via* SE, it is important to continue to accumulate information across many genotypes to support the development of propagation protocols for practical applications.

In the present case study in Niigata Prefecture, because field planting is restricted to the autumn season from September to November, the somatic plug plants that are transplanted into seedling containers in the spring, and reach the required size in the autumn of the same year, can be planted in the field as 1-year-old seedlings. The seedlings that did not reach the required height can be planted in the field in the following autumn as 2-year-old seedlings. Therefore, depending on seedling growth and conditioning, the cultivation period of the containerized seedlings in the nursery can vary from around 9 to 16 months.

**Figure 10:**
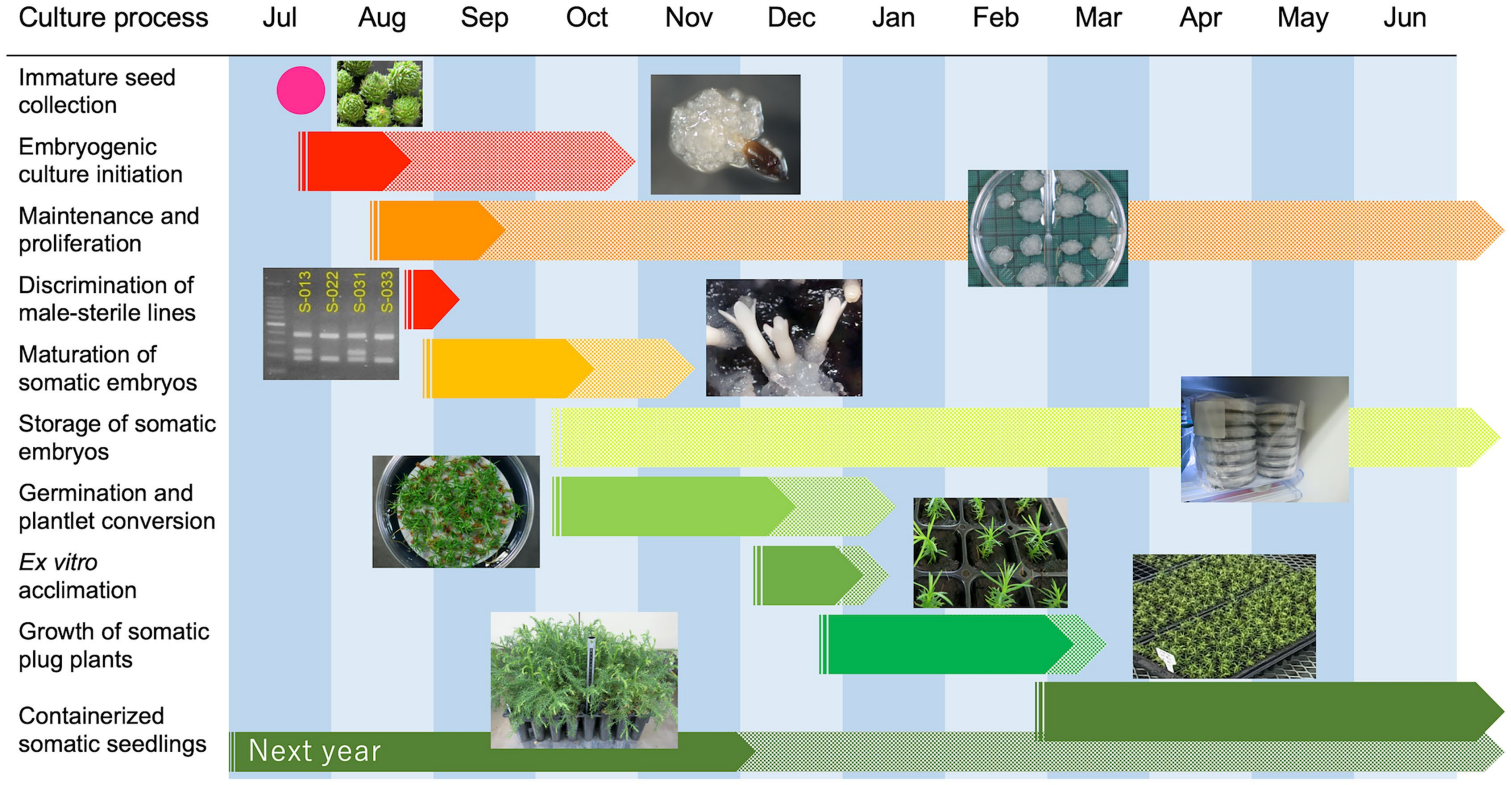
A representative example schedule for the propagation of pollen-free sugi (*C. japonica*) *via* somatic embryogenesis.

Finally, summarized conditions and results for each stage of our methodology are shown in Table 5. Additionally, a representative schedule for the propagation of pollen-free sugi *via* SE (a case study for Niigata Prefecture, central region of Japan) is presented as a reference for practical uses (Figure 10). This schedule can be adapted to other regions of the country by adjusting the steps according to the respective planting season. The schedule is somewhat rigid during the first year due to the fact that the onset of SE is limited to a very short period of approximately 1 month, in which the explant is highly responsive (mainly July throughout Japan); however, after the first year, when embryogenic cultures have already been established, the schedule can be adjusted according to the objective and nursery practices. Flexibility can further increase if somatic embryo storage is used to regulate the production of seedlings at the required time.

**Table 5:** Summarized conditions and results for each stage in the propagation of pollen-free sugi (*C. japonica*) plants *via* somatic embryogenesis.

Stages	Summarized conditions	Summarized results	References
Embryogenic culture initiation	Culture of megagametophyte explants (isolated from seeds collected in mid-July and containing immature zygotic embryos) on EM-1 medium^1^ for 1–3 months.	Initiation rate: 26.0% (SD ± 18.9); Initiation range: 0.9–49.9%	Maruyama et al., 2014, 2021a
Maintenance and proliferation of embryogenic cells	Subculture of induced embryogenic cells on EM-2 medium^1^ every 2 weeks.	Proliferation rate: 5.3 times (SD ± 1.0); Proliferation range: 3.5–7.5 times	This report
Discrimination of male-sterile embryogenic cell lines	DNA extraction from embryogenic cells by InstaGene and DNA marker diagnosis of *MS1* genotype.	Discrimination probability of male-sterile embryogenic cell lines: 100%	Tsuruta et al., 2021a
Maturation of somatic embryos	Culture of proliferated embryogenic cells on EM-3 medium^1^ for 6–10 weeks.	Average number of mature embryos per plate: 243.6 (SD ± 163.7); Range: 25.0–513.8	Tsuruta et al., 2021b
Storage of somatic embryos	Storage of developed somatic embryos on EM-3 medium^1^ at 5°C for 0–2 years.	Germination rate: 92.7% (SD ± 7.5); Germination range: 78.8–100%	This report
Somatic embryo germination and plantlet conversion	Culture of mature somatic embryos on EM-4 medium^1^ for 8–12 weeks.	Germination rate: 87.1% (SD ± 11.9); Germination range: 52.1–97.5%; Conversion rate: 84.8% (SD ± 12.6); Conversion range: 47.7–96.6%	Tsuruta et al., 2021b
*Ex vitro* acclimatization and growth of somatic plants	Acclimatization of somatic plantlets in plant plugs^2^ inside greenhouse with 20–30 s mist watering every 20–30 min during the daytime for 3–4 weeks. Then, plants were irrigated by manual watering once per day and fertilized with a plant-food solution^3^ once per week.	Survival rate: 77.6% (SD ± 12.1); Survival range: 63.9–87.0%. Plug plants with heights greater than 5 cm are ready to be transplanted into seedling containers in the spring season^4^	This report
Containerized somatic seedlings	Transfer of acclimatized plug plants into seedling containers^4^ kept in the nursery with 15–30 min daily sprinkler watering and once per week additional fertilization^5^ for 9–16 months.^6^	Survival rate: 91.7% (SD ± 7.4); Survival range: 89.6–100.0%. Containerized seedlings with heights greater than 30 cm are ready to be planted in the autumn season^4^	This report

1 See Supplementary Table S1.

2 200 holes per tray Jiffy Preforma^®^ plant plugs (Sakata Seed Co., Yokohama, Japan).

3 Peter’s 20-20-20 soluble fertilizer (The Hyponex Co., Inc., Hyponex Japan, Osaka, Japan).

4 JFA-150 (150 ml; 8 × 5 cavities per tray) seedling containers (National Federation of Forest Seedling Cooperatives, Tokyo, Japan).

5 Hyponex 6-10-5 liquid fertilizer (The Hyponex Co., Inc., Hyponex Japan, Osaka, Japan).

6 Niigata Prefecture case study.

## Conclusion

In this paper, we presented an improved and simplified methodology for the propagation of pollen-free sugi (*C. japonica*) by combining the early selection of male-sterility lines by MAS and somatic seedling production through SE. This methodology uses simple DNA extraction with InstaGene (as first reported for conifers), a direct marker to discriminate male-sterile lines at the undifferentiated EC stage, and an efficient large-scale somatic embryo production system, which altogether allow for the production of 100% pollen-free somatic sugi plants under a considerably shorter timeframe than from seeds or cuttings. On the other hand, our methodology for the collection of somatic embryos from maturation medium, embryo germination and plantlet conversion, and the subsequent *ex vitro* acclimatization in plant plugs allowed us to further simplify our previously established protocols. Furthermore, in this study, we demonstrated that mature somatic embryos can be stored for at least 2 years without a considerable loss in germination capacity, which provides flexibility to manage the somatic seedling production schedule. This methodology provides basic and practical information that will help to accelerate the production of pollen-free Japanese cedar plants across Japan.

## Data Availability Statement

The original contributions presented in the study are included in the article/Supplementary Material, further inquiries can be directed to the corresponding author.

## Ethics Statement

Written informed consent was obtained from the individual(s) for the publication of any potentially identifiable images or data included in this article.

## Author Contributions

TM, SU, MT, and YM: conceptualization and methodology. YM: funding acquisition and project administration. TM, MT, KK, and YB: plant material preparation. TM, MT, and SU: data curation. TM, MT, SU, KK, and YB: experiments and data analysis. TM and MT: writing – original draft. TM, MT, SU, KK, YB, and YM: writing – review and editing. All authors have read and agreed to the published version of the manuscript.

## Funding

This research was supported by grants from the Ministry of Agriculture, Forestry and Fisheries of Japan (MAFF) and Bio-oriented Technology Research Advancement Institution [BRAIN; The Science and Technology Research Promotion Program for Agriculture, Forestry, Fisheries and Food Industry (No. 28013B) and grants from BRAIN Research Program on Development of Innovative Technology (No. 28013BC)].

## Conflict of Interest

KK was employed by the company Verde Co., Ltd.

The remaining authors declare that the research was conducted in the absence of any commercial or financial relationships that could be construed as a potential conflict of interest. 

## Publisher’s Note

All claims expressed in this article are solely those of the authors and do not necessarily represent those of their affiliated organizations, or those of the publisher, the editors and the reviewers. Any product that may be evaluated in this article, or claim that may be made by its manufacturer, is not guaranteed or endorsed by the publisher.
