# Supplementary material for: An Improved and Simplified Propagation System for Pollen-Free Sugi (Cryptomeria japonica) via Somatic Embryogenesis
Source: Front Plant Sci. 2022 Feb 8;13:825340. doi: 10.3389/fpls.2022.825340 (PMC8861444; doi:10.3389/fpls.2022.825340)
Supplement: Supplementary file 1 [file Data_Sheet_1.docx]

Supplementary Material

**
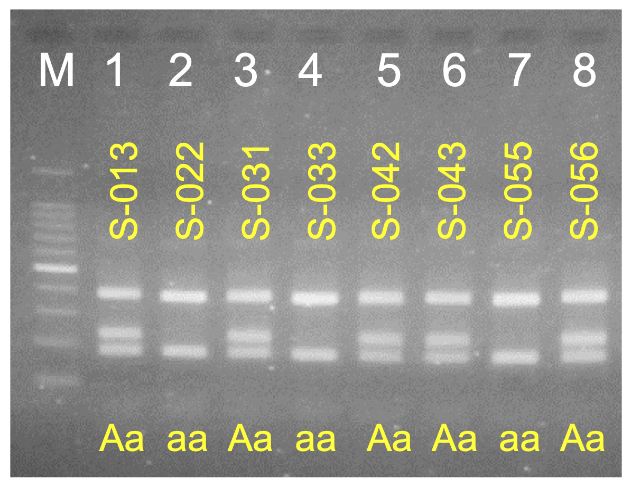
**

Com

WT

mut

**Supplementary Figure S1.** An example of the discrimination of male-sterile embryogenic cell lines by electrophoresis of *MS1* marker-assisted selection. The amplified PCR product consists of a common control band (Com: 385 bp and/or 381 bp) and the allele-specific band for the normal allele, namely wildtype (WT: 233 bp) and/or the mutant allele (mut: 183 bp), allowing *MS1* genotyping. M: 100 bp ladder marker, 1–8: somatic embryogenic lines (‘Shindai 3’ × ‘Suzu 2’).

**Supplementary Figure S2.** Somatic embryo maturation efficiency for different embryogenic cell lines of pollen-free sugi (*C. japonica*) derived from the ‘Shindai 3’ × ‘Suzu 2’ seed family (average number of cotyledonary embryos per plate ± SD for each cell line). The different letters indicate significant differences of the cell lines (*P* < 0.05, pairwise comparison with BH adjustment).

**Supplementary Table S1.** Culture media used in the propagation of pollen-free sugi (*C. japonica*) plants via somatic embryogenesis.

| **Components** | **Initiation** | **Maintenance/ Proliferation** | **Maturation** | **Germination/ Conversion** |
| --- | --- | --- | --- | --- |
|  | **EM-1 medium** | **EM-2 medium** | **EM-3 medium** | **EM-4 medium** |
| ***Basal salts* (mg/L)** |  |  |  |  |
| KNO_3_ | 500 | 500 | 1000 | 500 |
| MgSO_4_.7H_2_O | 250 | 250 | 500 | 250 |
| CaCl_2_.2H_2_O | 37.5 | 37.5 | 70 | 37.5 |
| Ca(NO_3_)_2_.4H_2_O | 30 | 30 | 60 | 30 |
| NaNO_3_ | 30 | 30 | 60 | 30 |
| KH_2_PO_4_ | 35 | 35 | 70 | 35 |
| NaH_2_PO_4_.2H_2_O | 80 | 80 | 160 | 80 |
| KCl | 40 | 40 | 80 | 40 |
| MnSO_4_.4H_2_O | 10 | 10 | 20 | 10 |
| H_3_BO_3_ | 20 | 20 | 40 | 20 |
| ZnSO_4_.7H_2_O | 12.5 | 12.5 | 25 | 12.5 |
| KI | 0.5 | 0.5 | 1 | 0.5 |
| CuSO_4_.5H_2_O | 1.2 | 1.2 | 2.4 | 1.2 |
| Na_2_MoO_4_.2H_2_O | 0.1 | 0.1 | 0.2 | 0.1 |
| CoCl_2_.6H_2_O | 0.1 | 0.1 | 0.2 | 0.1 |
| FeSO_4_.7H_2_O | 15 | 15 | 30 | 15 |
| NaEDTA | 20 | 20 | 40 | 20 |
| ***Vitamins* (mg/L)** |  |  |  |  |
| Thiamine HCl | 2.5 | 2.5 | 5 | 2.5 |
| Pyridoxine HCl | 0.25 | 0.25 | 0.5 | 0.25 |
| Nicotinic acid | 2.5 | 2.5 | 5 | 2.5 |
| Glycine | 2.5 | 2.5 | 5 | 2.5 |
| myo-Inositol | 500 | 500 | 1000 | 500 |
| ***Plant growth regulators* (μM)** |  |  |  |  |
| 2,4-D | 10 | 3 |  |  |
| BAP | 5 | 1 |  |  |
| Abscisic acid^1^ |  |  | 100 |  |
| ***Amino acids*^1^ (mg/L)** |  |  |  |  |
| Casein acid hydrolysate | 500 |  |  |  |
| Glutamine | 1000 | 1500 | 2000 |  |
| Asparagine |  |  | 1000 |  |
| Arginine |  |  | 500 |  |
| Citrulline |  |  | 79 |  |
| Ornithine |  |  | 76 |  |
| Lysine |  |  | 55 |  |
| Alanine |  |  | 40 |  |
| Proline |  |  | 35 |  |
| ***Other additives* (mg/L)** |  |  |  |  |
| Sucrose | 10000 | 30000 |  | 20000 |
| Maltose |  |  | 30000 |  |
| Polyethylene glycol 6000 |  |  | 175000 |  |
| Activated charcoal |  |  | 2000 | 2000 |
| Gellan gum | 3000 | 3000 | 3300 |  |
| Agar |  |  |  | 10000 |
| ***pH*** | 5.8 | 5.8 | 5.8 | 5.8 |

^1^ Filter-sterilized stock solution was added after autoclaving and cooling to 60°C–65°C.

**Supplementary Table S2.** Germination and plantlet conversion of pollen-free sugi (*C. japonica*) somatic embryos derived from different embryogenic cell lines (ECLs) of the ‘Shindai 3’ × ‘Suzu 2’ seed family.

| **Cell line** | **Replicates** | **Germination rate (%)** | | |  | **Conversion rate (%)** | | |
| --- | --- | --- | --- | --- | --- | --- | --- | --- |
|  |  | **Mean** | **SD** | **Significance^1^** |  | **Mean** | **SD** | **Significance^1^** |
| SSD-009 | 5 | 74.1 | 20.3 | d |  | 72.2 | 18.1 | d |
| SSD-018 | 10 | 97.5 | 3.4 | a |  | 96.6 | 4.6 | b |
| SSD-029 | 10 | 91.6 | 17.8 | cf |  | 89.3 | 18.5 | af |
| SSD-070 | 5 | 88.9 | 14.5 | ef |  | 85.9 | 17.2 | ef |
| SSD-073 | 10 | 93.6 | 5.3 | ab |  | 91.6 | 6.1 | abc |
| SSD-100 | 10 | 94.4 | 3.9 | af |  | 92.2 | 5.1 | cf |
| SSD-111 | 5 | 52.1 | 10.0 | g |  | 47.7 | 11.8 | h |
| SSD-113 | 10 | 89.6 | 7.8 | ce |  | 88.1 | 8.5 | ef |
| SSD-137 | 6 | 94.9 | 1.9 | acf |  | 93.0 | 3.1 | bf |
| SSD-168 | 5 | 63.4 | 10.9 | g |  | 61.3 | 11.9 | g |
| SSD-174 | 5 | 91.5 | 5.6 | bef |  | 88.3 | 8.4 | ef |
| SSD-182 | 10 | 94.2 | 4.3 | bcf |  | 92.7 | 5.7 | cf |
| SSD-183 | 5 | 91.4 | 6.6 | ef |  | 90.6 | 6.6 | cef |
| SSD-270 | 5 | 79.0 | 10.0 | d |  | 73.9 | 14.6 | d |
| SSD-272 | 5 | 87.2 | 9.0 | de |  | 84.8 | 8.9 | de |
| SSD-345 | 6 | 94.1 | 5.5 | bcf |  | 91.4 | 6.7 | cf |
| SSD-352 | 10 | 91.4 | 7.7 | ef |  | 90.6 | 7.7 | cf |
| SSD-377 | 5 | 89.8 | 10.7 | ef |  | 85.5 | 13.3 | ef |
| SSD-383 | 6 | 97.0 | 2.8 | ab |  | 95.3 | 3.1 | bc |

^1^ Significance of cell line difference is indicated by different letters (*P* < 0.05, pairwise comparison with BH adjustment).
